# Relevance of Intestinal Microbiota in Immunoglobulin A Vasculitis With Abdominal Involvement

**DOI:** 10.3389/fped.2022.943267

**Published:** 2022-07-14

**Authors:** Linqian Zhang, Xinyi Jia, Panjian Lai, Kang Wang, Yunguang Bao, Xiaobing Li

**Affiliations:** ^1^Jinhua Maternal and Child Health Care Hospital, Jinhua Women’s and Children’s Hospital, Jinhua, China; ^2^Children’s Hospital, Zhejiang University School of Medicine, Hangzhou, China

**Keywords:** immunoglobulin A vasculitis with abdominal involvement, intestinal microbiota high-throughput sequencing, *Veillonella*, *Ruminococcus*, *Bifidobacterium*, *Bacteroides*

## Abstract

**Background:**

We explored the intestinal microbiota changes in IgAV with abdominal involvement (IgAV-GI) at the acute and convalescent stages and evaluated the role of intestinal microbiota in the clinical course of patients with IgAV.

**Methods:**

A total of 37 patients with IgAV were included, and the control group comprised 37 age- and sex-matched healthy children. Stool samples were collected from 28 children with IgAV-GI (19 in the acute stage and 9 in the recovery stage) and from nine children with non-abdominal involvement. Fecal specimens were selected and DNA was obtained using an extraction kit which was then subjected to high-throughput sequencing and analysis.

**Results:**

There was no significant difference in the community structure of the intestinal microbiota among the IgAV-GI acute, IgAV-GI convalescence, and IgAV-non-GI stages. The abundance of *Veillonella* in the acute stage of IgAV-GI was significantly higher than that in IgAV-non-GI and convalescence stages, and *Ruminococcus* was the most abundant in IgAV-GI convalescence. The α-diversity of children with IgAV was significantly lower than that of healthy children, and healthy children had higher intestinal microbiota richness and more evenly distributed species. In terms of changes in intestinal microbial diversity in patients with IgAV at the genus level, obligate anaerobes such as *Bifidobacterium, Prevotella, Coprobacter, Prevotella_9, Blautia, Romboutsia, Parabacteroide, Subdoligranulum*, and *Roseburia* were significantly reduced, and the enrichment of facultative anaerobe was represented by *Bacteroides, Lachnoclostridium*, and *Alistipe*.

**Conclusion:**

Different bacterial species may be involved in the pathogenesis of different types of IgAV-GI. Differences were observed in the intestinal microbiota between healthy children and children with IgAV.

## Introduction

Immunoglobulin A vasculitis (IgAV), an IgA-mediated vascular inflammatory disease occurs in 8–20 out of 100,000 children every year, of which approximately 50% are children under 5 years. It mainly affects the skin (100%), digestive tract (50–75%), joints, and kidneys. Clinically, it can be divided into five types: simple, abdominal, kidney, joint, and mixed. Abdominal hypersensitivity is associated with abdominal pain, abdominal distension, distension of the heart, vomiting, hematochezia, melena, and other gastrointestinal symptoms such as purpura, accounting for approximately 2–3 cases (1). There are still many unclear aspects regarding the causes and pathophysiology of IgAV. The IgA immune complex is produced by reactions to exogenous substances, such as bacteria (group A streptococcal infections in particular), viral infections, chemical agents, certain foods, insect bites, vaccines, and deposits on small-vessel walls. These substances activate the complement system by deposition of the IgA immune complex on the vessel walls, resulting in the production of cytokines and IgAV symptoms (2). Many studies in China as well as abroad have suggested that the composition of the intestinal microbiota is related to allergic diseases, among which the influence of immune imbalance caused by intestinal microbiota should not be ignored, as the human body is interdependent and symbiotic community with bacteria. A large number of bacteria are distributed on the skin surface, gastrointestinal tract, oral cavity, respiratory tract, urogenital tract, and feces. In particular, there is a great variety and number of gastrointestinal bacteria, accounting for approximately 80% of the total microbial population in the human body. Studies have shown that the intestinal microbiota is an important factor regulating the immune system response, and immunoregulatory factors such as interleukins are laboratory parameters associated with disease recurrence (3, 4). An increasing number of reports and recurrences of IgAV, and recent studies on the immune mechanism of IgAV have found that changes in intestinal microbiota may affect the development and prognosis of IgAV. The purpose of this study was to explore difference in the intestinal microbiota in children with IgAV vs. healthy children. The changes in the intestinal microbiota in children with IgAV-GI before and after treatment and the comparison with children with IgAV-non-GI, as well as whether the structure of the intestinal microbiota affects the recurrence of IgAV, were investigated to provide a theoretical basis for exploring the regulation of intestinal microbiota by probiotics and other drugs to treat and improve the prognosis of IgAV.

## Materials and Methods

### Materials

A total of 37 children aged 3–14 years newly diagnosed with IgAV at the pediatric nephrology–rheumatology unit of a tertiary-care hospital between 2018 and 2020 were selected. The IgAV diagnoses were made in children under 14 years of age according to the criteria set by EULAR/PRINTO/PRES (5). Thirty-seven age- and sex-matched healthy children who attended outpatient clinics were included in the control group. The two groups were compared (Group A: IgAV; Group B: Control healthy group). Group A was further divided into abdominal type (GI involvement diagnosed depending on clinical gastrointestinal symptoms and endoscopic changes) according to whether there were abdominal clinical symptoms (acute stage was group A, 19 cases); after treatment and the symptoms were relieved within 3 days (group B, 9 cases); and non-abdominal type [group C (acute stage of lgAV-non-GI) comprised 23 cases]. A total of 42 fecal specimens were collected. The study protocol was approved by the local ethics committee, and informed parental consent was obtained in each case. Exclusion criteria included patients who had used antibiotics in the 3 months prior to the IgAV diagnosis; the presence of additional gastrointestinal, kidney, or rheumatologic disease; diagnosis of another systemic vasculitis; incomplete clinical/laboratory data; and absence of follow-up visits.

### Methods

#### Data Collection

Patient files were evaluated in terms of demographic data (age at presentation, sex, height, and weight), system involvement (palpable purpura, arthritis/arthralgia, and gastrointestinal, and/or kidney involvement), recovery time of symptoms with regard to the involved systems, treatment, disease period, and follow-up time.

#### Collection of Specimens

Fecal specimens were collected from children with IgAV before treatment in the acute and chronic phases, and each member was given a stool collection kit containing a 10 ml plastic feces specimen jar, gloves, a study information sheet, and written instructions for providing a sample. Patients were asked to self-collect a fresh fecal sample the following day with sterile EP tubes, which were then stored in a freezer at -80^°^C for later use.

#### DNA Extraction and High-Throughput Sequencing

Total bacterial DNA of fecal samples was extracted using a DNA stool test box (Omega-soil DNA kit, Omega Bio-tek, United States), and high-throughput 16S rRNA gene sequencing was performed. A sequencing library was used to construct quality control and high-throughput sequencing.

#### Statistical Analysis

The IBM SPSS statistics 21 software was used for descriptive statistical analysis of the data, mainly to describe the statistical sample data, such as age, gender, and laboratory test results and the data conforming to a normal distribution were expressed as means ± the standard deviation, and enumeration data were expressed as percentages. The Kruskal–Wallis test was applied to data that did not conform to a normal distribution. The *p* < 0.05 was accepted as significant.

## Results

### General Information

According to the inclusion criteria, a total of 37 children with IgAV were included in this study, with an average age of (7.35 ± 3.11) years, comprising 22 men and 15 women. Of these, 19 patients with abdominal pain, vomiting, and other digestive tract symptoms were included in the group of IgAV-GI, and all the 19 patients (10 men and 9 women) underwent endoscopy. Electronic gastroscopy examination showed that the mucosa of the stomach and duodenum exhibited different degrees of erosion, scattered in different sizes of bleeding spots. The mucosa was brittle and readily bled when touched. The gastric antrum, duodenal sphere, and descending part were all involved (2 cases) along with the descending part of the duodenum (6 cases). There were only a few bleeding points in the stomach, or congestion and edema, in the gastric antrum (11 cases). The esophageal mucosa was normal, which was consistent with the changes in the gastrointestinal mucosa of the IgAV-GI (6). Group A (acute stage of IgAV-GI) comprised 19 cases, Group B (recovery stage of IgAV-GI) comprised 9 cases, and Group C (acute stage of IgAV-non-GI) comprised 23 cases. A total of 42 fecal samples were collected. All children with IgAV received symptomatic supportive treatment, and some were treated with hormones to suppress the inflammatory response. The clinical symptoms disappeared and improved, and the average hospital stay was 7.43 ± 4.21 days. The chi-square test was used to assess the gender differences among the three groups (χ^2^ = 2.308, *p* = 0.3152), and a univariate variance test was used to assess the age differences (χ^2^ = 0.753, *p* = 0.4791). There were no significant differences in sex or age composition between the three groups.

### Comparison of the Fecal Microbiota Between Children With Immunoglobulin A Vasculitis and Healthy Children

#### Diversity Analysis (α和β)

The α-diversity of the children with IgAV was significantly lower than that of healthy children, which was reflected in the smaller community richness and lower diversity (Table 1; *t* < 0.05).

**TABLE 1:** Comparison of the α-diversity between the groups.

	Community richness index			Community diversity index	
	Chao	Ace	Observed species (OTU)	Simpson	Shannon
lgAV	125	125	120	0.9	4.75
Control group	346	346	252	0.92	4.9

**FIGURE 1:**
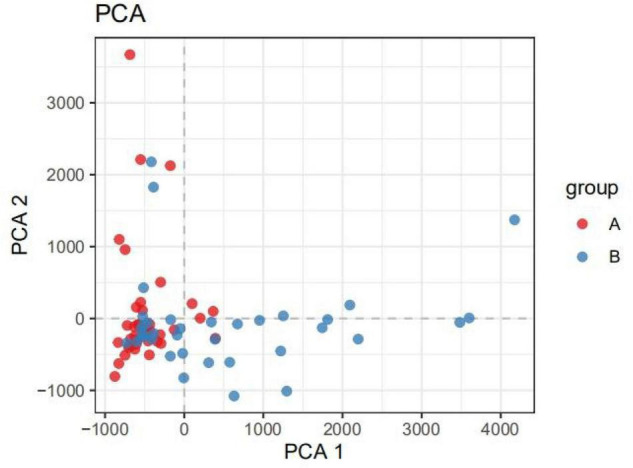
PCA analysis diagram. (Dots of different colors or shapes represent different groups of samples; the closer the two sample points are, the more similar the species composition of the two samples is).

The β-diversity of the children indicated that there were significant differences in the intestinal microbiota composition between children with IgAV and healthy children (Figure 1).

#### Structural Difference Analysis

**FIGURE 2:**
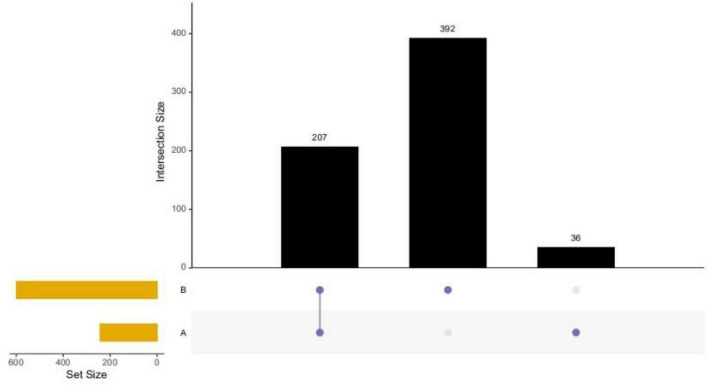
Full-level species Upset plot. (Group A: IgAV; Group B: healthy controls).

**FIGURE 3:**
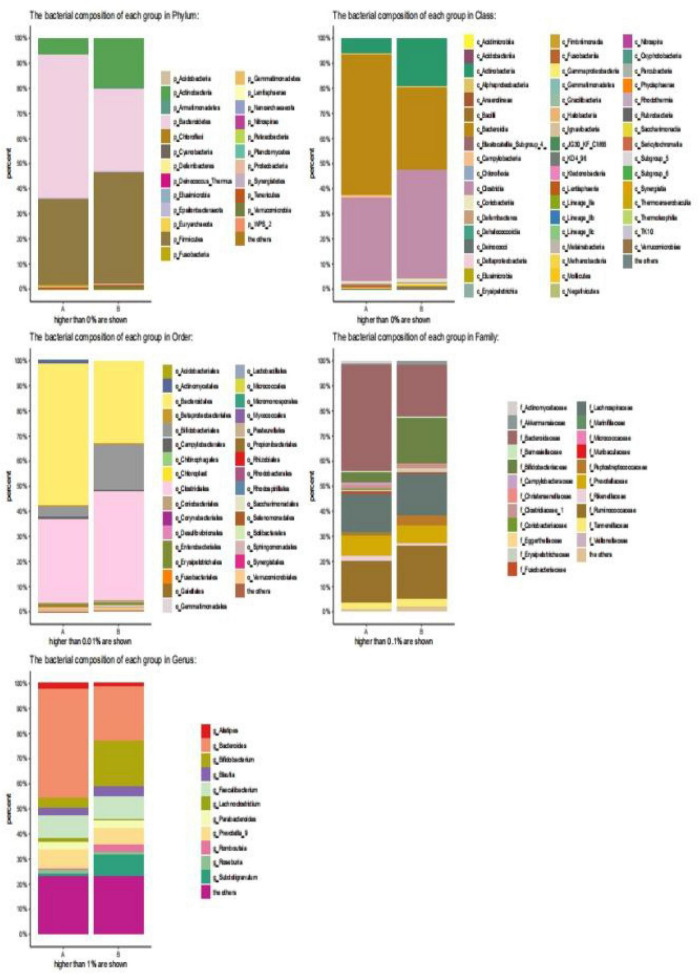
Bar diagram of community. (The abscissa is the grouping of sample attribution, the ordinate is the proportion of species in the grouping, the column of different colors represents different species, and the length of the column represents the proportion of the species).

Healthy children had higher intestinal microbiota richness (Figure 2). The bacterial composition of each group at different levels (e.g., phylum, class, order, family, and genus) is shown in Figure 3. At the phylum level, Bacteroidetes (55%) were the dominant bacteria in the IgAV group, whereas Firmicutes (45%) and Actinomycetes (20%) were the dominant bacteria in the healthy control group.

At the class level, Bacteroidetes (56%) were the dominant bacteria in the IgAV group, whereas Clostridium (43%) and Actinomycetes (20%) were the dominant bacteria in the healthy control group.

At the order level, Bacteroides (58%) were the dominant bacteria in the IgAV group, whereas Clostridium (44%) and Bifidobacterium (18%) were the dominant bacteria in the healthy control group.

At the family level, *Bacteroidaceae* (43%) were the dominant bacteria in IgAV, whereas *Bifidobacteriaceae* (17%) and Rumen *Bacteriaceae* (20%) were the dominant bacteria in the healthy control group.

At the genus level, *Bacteroides* (45%) were the dominant bacteria in IgAV, whereas *Bifidobacterium* (21%) and *Subdoligranulum* (8%) were the dominant bacteria in the healthy control group.

#### Species Difference Analysis

**FIGURE 4:**
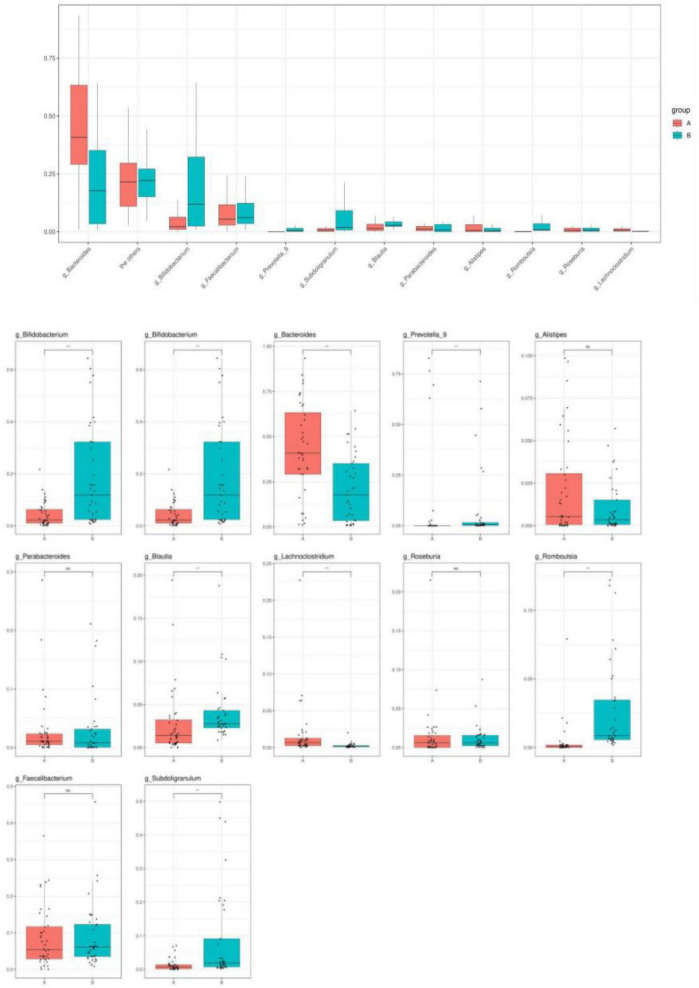
Species with abundance greater than 0.1% at the genus level.

Changes in intestinal microbial diversity in patients with IgAV at the genus level included significant reduction of the obligate anaerobes: *Bifidobacterium*, *Prevotella*, *Coprobacter*, *Prevotella_9*, *Blautia*, *Romboutsia*, *Parabacteroide*, *Subdoligranulum*, and *Roseburi*a. The enrichment of facultative anaerobes was represented by *Bacteroide*s, *Lachnoclostridium*, and *Alistipe* (Figure 4).

### Comparison of Fecal Microbiota Between Acute Stage (A) and Recovery Stage in Children With Immunoglobulin A Vasculitis With Abdominal Involvement (B) and Non-GI type (C)

#### Diversity Analysis(α和β)

**FIGURE 5:**
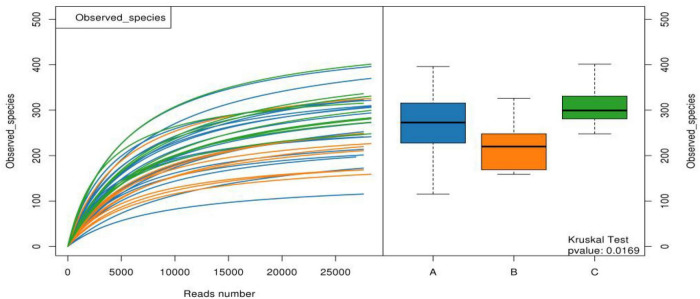
Observed species of the three groups of samples. (A: acute stage of IgAV-GI, B: recovery stage of IgAV-GI, C: IgAV-non-GI).

The α-diversity observed in the species of samples from group B was significantly lower than those from groups A and C, indicating that the species richness of group B was lower than that of groups A and C, as shown in Figure 5. The other Chao1, Goods Coverage, Shannon, and Simpson index differences were not statistically significant (Kruskal–Wallis test, *p* > 0.05).

**FIGURE 6:**
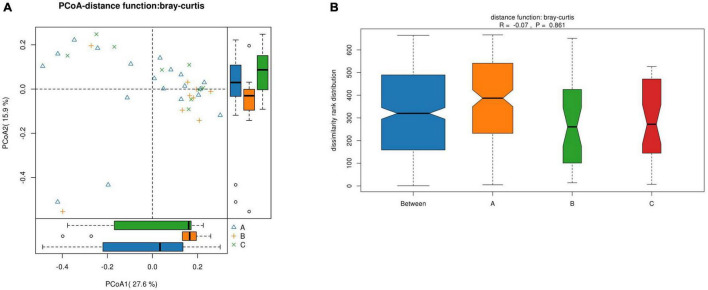
The β-diversity of the intestinal microbiota of the children in the three groups. **(A)** Comparison of the similarity of species between samples by the distance between the samples in the figure; the greater the distance, the greater the difference. **(B)** Between represents the difference between groups, while the other items are within groups. The greater the distance, the greater the difference, and the thickness represents the sample size).

For the β-diversity, there was no significant difference in intestinal microbiota formation among the different groups of children with IgAV-GI (*r* = –0.07, *p* = 0.8610), as shown in Figure 6.

#### Structural Difference Analysis

**FIGURE 7:**
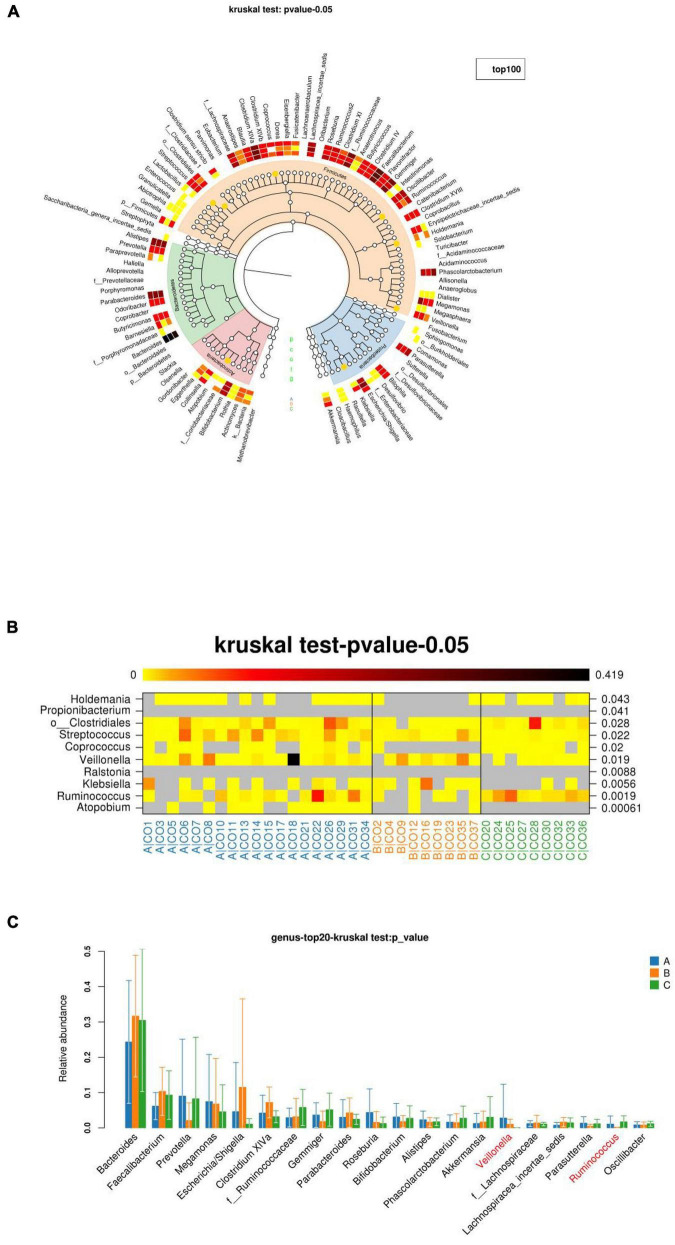
The richness and microbiota structure of the intestinal dominant bacteria in the three groups. **(A)** Innermost layer is the species classification tree, which contains three types of bottoms used with “gates” on top of color labeling. The orange dots indicate significant differences between the three groups (*Q*-values), with a layer of red in the middle. White and white are the average richness heat map, and the darker the color, the greater the abundance. The outermost layer is the injection interpretation of the species. **(B)** Diagram of the richness of different species. The darker the color, the higher the richness. The left side shows the corresponding species annotation, and the right side shows the *p*- or *Q*-values of the statistical test. **(C)** Top 20 species richness microbiota of the three groups of samples at the species level).

Groups A, B, and C can be seen in the species classification tree (Figure 7A) and chart of different species (Figure 7B). There were significant differences in the species composition, and the top 20 species richness microbiota of the three groups of samples were compared (Figure 7C). The non-parametric Kruskal–Wallis test was used for the three groups of samples, and the differences were statistically significant. The relative abundance of *Veillonella* in group A was significantly higher than that in group B and group C. The relative abundance of Ruminococcus was the highest in group B (p = –0.0496).

## Discussion

### Differences in Fecal Microbiota Between Immunoglobulin A Vasculitis and Healthy Children

The α-diversity of children with IgAV was significantly lower than that of healthy children, and healthy children had higher intestinal microbiota richness and more evenly distributed species. The β-diversity of the children indicated that there were significant differences in the intestinal microbiota compositions between children with IgAV and healthy children. Changes in intestinal microbial diversity in patients with IgAV at the level of phylum, class, order, family, and genus indicated that *Bacteroides* were the dominant bacteria. The numbers of obligate anaerobes, such as *Bifidobacterium*, *Prevotella*, *Coprobacter*, *Prevotella_9*, *Blautia*, *Romboutsia*, *Parabacteroides*, *Subdoligranulum*, and *Roseburia*, were significantly reduced. Enrichment of facultative anaerobes was represented by *Bacteroides*, *Lachnoclostridium*, and *Alistipe*.

### Differences in Intestinal Microbiota Between the Acute Stage and the Convalescent Stage of Immunoglobulin A Vasculitis With Abdominal Involvement

The intestinal microbiota of *Veillonella* in children with IgAV-GI in the acute stage was significantly higher than that in children with convalescent stage and IgAV-non-GI. *Veillonella* is a gram-negative anaerobic micro pellebacterium that belongs to the Firmicutes phylum, which is involved in the composition of the normal microbiota in the oral cavity, urogenital tract, respiratory tract, and intestinal tract of humans and animals. The risk factors for infection include periodontal disease, immune deficiency, intravenous drug use, and premature birth (7). Epidemiological data show that it is a common oral pathogen that can readily cause dental caries, dental pulp infection, and periodontitis (8). Other serious infections include meningitis, epidural fluid, osteomyelitis, human infection, infected pleural inflammation, endocarditis, and mycohememia (8–10). Some studies have reported that *Veillonella* is associated with poor oral health, a high body mass index, and older age (11). Gastroesophageal reflux has also been shown to promote the formation of Barrett’s esophagus (12). It is seldom known at the molecular level why these associations exist (8), and it has been speculated that the increased concentration of *Veillonella* in IgAV-GI maybe a risk factor for the involvement of the digestive tract. The abundance of *Ruminococcus* increased in the recovery period of IgAV-GI. A study has shown that reduced abundance of *Ruminococcaceae* (*Ruminococcus* genus), reduced levels of butyrate and propionate production, and reduced anti-inflammatory activity of short-chain fatty acids (SCFAs) resulted in abnormal Th2 immune responses, and ultimately eczema. In infants without allergies, the phase pair abundance of *Ruminococcaceae* was negatively correlated with IL-6 and TNF-α induced by Toll-like receptor-2 (TLR-2), and the lower relative abundance of *Ruminococcaceae* appeared to be related to food sensitization. It can also predict the development of atopic eczema. It has also been proven recently that active *Ruminococcaceae* produces isocholic acid, and the detoxification pathway of isocholic acid affects the growth of a main genus of bacteria, namely *Bacteroides*, in human gut (13). A causal relationship has been reported between the decreased abundance of *Lachnospira*, *Veillonella*, *Faecalibacterium*, and Rosia in early infancy and an increased risk of asthma (14). *Ruminococcus* occupies a dominant position in the intestinal tract of infants and its level in water is as high as that of *Bifidobacterium*. They share the same metabolic pathway, involving the degradation of sugar and mucin, and the sugar released by mucin degradation is conducive to the growth of other bacteria (15–17). The known physiological functions of the human intestinal microbiota, ranging from protective functions to metabolic regulation, are mainly related to the production of SCFAs. SCFAs are produced as intestinal microbiota metabolites and they have a major impact on the host as an energy source, regulating the development of immune cells, and inhibiting inflammation as regulators of gene expression and signal molecules recognized by specific receptors. Three of the main SCFAs, acetate, propionate, and butyrate, differ significantly in their potential effects on the host physiology. First, their functions and tissue distributions differ. Butyrate is preferred as an energy source of the intestinal mucosa, which helps liver gluconeogenesis and acetate in the blood to reach the highest systemic concentration. Butyrate also protects against colorectal cancer, promotes satiety, and lowers cholesterol levels in conjunction with propionate. Acetate is the net fermentation product of most intestinal anaerobes. It is also produced by the reduction of acetic acid and is the main component of SCFAs in the intestinal cavity. On the other hand, propionate and butyrate are produced by different subgroups of gut bacteria. Butyric acid is mainly produced by Firmicutes, such as *Ruminococcaceae*, *Lachnospiraceae*, *Erysipelotrichaceae*, and *Clostridiaceae* (18). *Veillonella* does not produce butyric acid, which further suggests that low levels of butyric acid production in the intestinal microbiota, which can lead to low levels of butyrate, may be a significant risk factor for IgAV-GI infection. Due to the recovery and abundance of *Clostridium*, the content of butyric acid production in the intestinal microbiota increases, the level of butyrate increases, and the intestinal immune regulation function recovers, leading to the disappearance of abdominal symptoms of IgAV. In addition, in the IgAV-GI, relative to the abdomen type, *Ruminococcaceae* bacteria genus richness reduces the increase in the genus *Veillonella*. This suggests there may be a low level of butyrate and that intestinal barrier function is impaired, leading to the abdominal symptoms associated with the occurrence of IgAV-GI.

## Conclusion

This study revealed the importance of microbiota imbalances in IgAV. Compared with healthy children, the intestinal microbiota diversity of IgAV patients was altered, mainly manifesting as decreased α-diversity, decreased intestinal microbiota richness, and uneven distribution. At the level of phylum, class, order, family, and genus, Bacteroides were the dominant bacteria. The number of obligate anaerobes, such as *Bifidobacterium*, *Prevotella*, *Coprobacter*, *Prevotella_9*, *Blautia*, *Romboutsia*, *Parabacteroide*, *Subdoligranulum*, and *Roseburia*, was significantly reduced. The enrichment of facultative anaerobes was represented by *Bacteroides*, *Lachnoclostridium*, and *Alistipe*. It also appears that decreased *Ruminococcaceae* and increased *Veillonella* may promote the incidence of IgAV-GI through a decrease in the level of the metabolite butyric acid. These results further enhance the possibility of using microbial agents as a basic treatment for IgAV-GI in the future, so as to improve the clinical treatment effect, prevent the disease progression, and even possibly achieve prevention. Due to the small sample size of this study, the interference of other factors in the treatment process, and the possibility of single-center sample bias further studies are still needed.

## Data Availability Statement

The original contributions presented in this study are included in the article/supplementary material, further inquiries can be directed to the corresponding author.

## Ethics Statement

The studies involving human participants were reviewed and approved by the Ethics Committee of Jinhua Central Hospital, Zhejiang Province. Written informed consent to participate in this study was provided by the participants or their legal guardian/next of kin.

## Author Contributions

XL: conceptualization. LZ: writing—original draft preparation. PL: methodology. KW: data curation. YB and XJ: writing—review and editing. XL: project administration. All authors have read and agreed to the published version of the manuscript.

## Conflict of Interest

The authors declare that the research was conducted in the absence of any commercial or financial relationships that could be construed as a potential conflict of interest.

## Publisher’s Note

All claims expressed in this article are solely those of the authors and do not necessarily represent those of their affiliated organizations, or those of the publisher, the editors and the reviewers. Any product that may be evaluated in this article, or claim that may be made by its manufacturer, is not guaranteed or endorsed by the publisher.
